# Development of Wearable Textile MIMO Antenna for Sub-6 GHz Band New Radio 5G Applications

**DOI:** 10.3390/mi15050651

**Published:** 2024-05-15

**Authors:** Pendli Pradeep, Mohammed Mahaboob Basha, Srinivasulu Gundala, Javed Syed

**Affiliations:** 1 Department of Electronics and Communication Engineering, Sreenidhi Institute of Science and Technology (Autonomous), Hyderabad 501301, India; pradeeppendli@gmail.com (P.P.); mmehboobbasha@gmail.com (M.M.B.); 2 Department of Electronics and Communication Engineering, Lakireddy Bali Reddy College of Engineering, N T R Dt., Mylavaram 521230, India; 3 Department of Mechanical Engineering, College of Engineering, King Khalid University, Abha 61421, Saudi Arabia; 4 Center for Engineering and Technology Innovation (CETI), College of Engineering, King Khalid University, Abha 61421, Saudi Arabia

**Keywords:** MIMO, jeans, irregular octagon, return loss, substrate, HFSS

## Abstract

In this paper, an irregular octagonal two-port MIMO patch antenna is designed specifically for New Radio (NR) 5G applications in the mid-band sub-6 GHz. The proposed antenna comprises an irregularly shaped patch antenna equipped with a regular 50-ohm feed line and a parasitic strip line antenna, and is partially grounded. Jeans material serves as a substrate with an effective dielectric constant of 1.6 and a thickness of 1 mm. This material is studied experimentally. The proposed antenna design undergoes analysis and optimization using the ANSYS HFSS tool. Furthermore, the design incorporates the influence of the slot on both the ground plane and the parasitic strip line to optimize performance, enhance isolation, and improve impedance matching among antenna elements. The dimensions of the jeans substrate are 40 mm × 50 mm. The simulated impedance bandwidth ranged from 3.6 GHz to 7 GHz and the measured bandwidth was slightly narrower, from 4.35 GHz to 7 GHz. The simulation results demonstrated an isolation level greater than 12 dB between antenna elements, while the measured results reached 28.5 dB, and the peak gain for this proposed antenna stood at 6.74 dB. These qualities made this proposed antenna suitable for various New Radio mid-band 5G wireless applications within the sub-6 GHz band, such as N79, Wi-Fi-5/6, V2X, and DSRC applications.

## 1. Introduction

The utilization of the multiple-antenna approach has been widely adopted in contemporary times. In particular, patch antennas have gained renown due to their simplistic design and exceptional performance in mobile communication systems. Over the past few decades, antennas have assumed a paramount role in wireless communication systems. Notably, extensive research efforts have demonstrated that employing multiple antennas, as opposed to a solitary unit, yields improvements in overall system performance encompassing data throughput, security, and transmission efficiency between transmitters and receivers. The past few decades have witnessed prolific research endeavors and a proliferation of publications in this field, resulting in the rapid emergence of a variety of antenna systems. Various New Radio mid-band 5G wireless applications within the sub-6 GHz band have been presented, such as N79 (4.4–5 GHz), Wi-Fi-5/6 (5.15–5.85 GHz/5.925–7.125 GHz), and V2X/DSRC (5.85–5.90 GHz) applications.

In [1], an innovative design for an extremely compact and wideband monopole antenna was described. This antenna is constructed on an elastic substrate made of Kapton-polyimide, and the radiating element is contained in a square plane. It comprises two rhombic elements. Attaining a wide bandwidth is essential to overcoming potential performance limitations when using wearable technology on the body. We therefore created a MIMO antenna designed specifically for wearable devices which has both a large spectrum and low mutual interference; using jeans fabric as its base provides optimal efficiency and compatibility when wearing it. To improve the performance of antennas, two I-shaped structures were joined via adhesive copper sheets that were fixed on a dielectric substrate in the two patches [2]. Recently, a customized microstrip patch antenna specially designed for 5G was designed by using polyethylene terephthalate, and the thickness of the antenna substrate was 0.125 mm [3]. A novel technique was presented to create Global Positioning System (GPS) patch antennas by gluing adhesive copper tape directly onto jeans [4]. This research examined the development of wearable antennas which included dual-band (UWB) single-band (SC) and meta material variants for maximum efficiency [5]. As mentioned in [6], the forward-thinking approach to research led to the development of an ant-fabric MIMO antenna specifically designed to be workable [7]. In [8], the need for an effective MIMO solution with minimal mutual coupling between its constituent antenna elements was highlighted. In the literature, various papers have attempted to understand the function of wearable antennas under different conditions for various applications [9,10,11,12,13,14,15,16]. In [17], a dual-band antenna was presented for 5G NR applications. SAR analysis for dual-band wearable antenna for WLAN was presented in [18]. In [19], wearable textile antennas utilizing ultra-wideband (UWB) technology were created to strengthen wireless body area networks for use with telemedicine and mobile health systems. In [20], an innovative textile multiple input multiple output (MIMO) antenna design inspired by reflective artificial surfaces (RIS) and electromagnetic band gap gaps (EBG) was introduced, made using viscose wool felt materials.

This paper describes an irregular octagonal two-port MIMO patch antenna developed for use with 5G wireless applications in the sub-6 GHz band. It comprises an irregular octagon-shaped patch antenna incorporating a 50-ohm feedline, a parasitic strip line, and partial grounding [21,22,23,24]. The simulated impedance bandwidth ranged from 3.6 GHz to 7 GHz (64%, while the measured impedance bandwidth covered 47% (4.35 GHz to 7GHz). The simulation results demonstrated isolation between antenna elements exceeding 12 dB in simulations and reaching 28.5 dB during measurements, and the peak gain for our proposed antenna stood at 6.74 dB. This paper’s structure is as follows: Section 1 introduces wearable antennas, Section 2 details the antenna design methodology, Section 3 presents the results, and Section 4 conducts parametric analyses. before concluding our paper in Section 5.

## 2. Antenna Design Method

**Figure 1:**
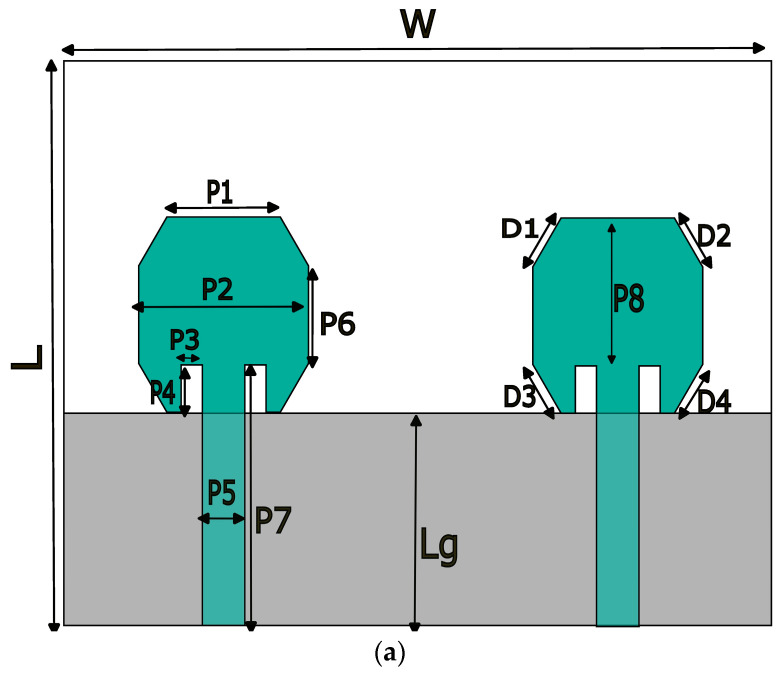

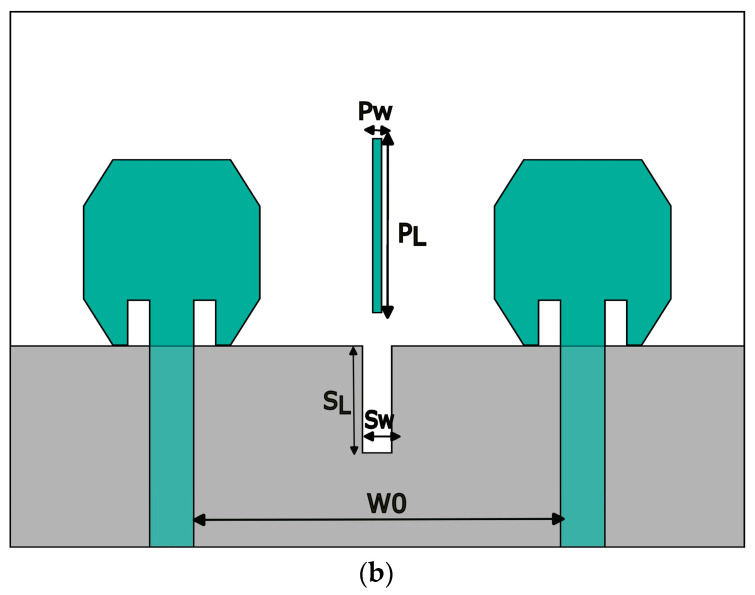
Proposed two-port irregular octagon-shaped MIMO antenna. (**a**) Conventional. (**b**) Proposed.

Figure 1 depicts a proposed antenna configuration as its top and back surfaces, respectively. This antenna features an irregular octagonal MIMO patch antenna configuration without any slots. This antenna is built upon an economical jeans substrate and powered through a 50-ohm microstrip line, featuring jeans fabric with an ideal loss tangent of 0.02 and relative permittivity of 1.6, as measured through loss tangent measurement and relative permittivity analysis. The dimensions for the antenna are 40 mm × 50 mm, while our investigations also explore the ground plane slotting effects as well as the parasitic strip lines between individual elements of its configuration, utilizing an ANSYS HFSS tool for analysis and fine-tuning purposes. The dimensions of the irregular octagonal patch are approximately equal to a quarter wavelength at operating frequency, i.e., 0.25λ at 4.6 GHz frequency, as shown in Equation (1), is used to find the resonant frequency and dimensions of the antenna.

(1) $$f_{r}\approx \frac{c}{4\left(P4+P8\right)\sqrt{\frac{1+\varepsilon_{r}}{2}}}$$

Here, c is the speed of light,${\;\varepsilon }_{r}$ is the relative permittivity, and *P*4 and *P*8 are the dimensions of the irregular octagonal patch.

A narrow slot is strategically incorporated into the ground plane to optimize antenna performance by altering the electric length of the current density distribution in the partial grounding. The investigation delves into the inter-element interactions, considering the inherent challenges associated with the two-port MIMO antenna design. A parasitic metallic strip line is located between the two irregular octagon-shaped patch antenna elements to mitigate interference and to provide requisite isolation. The antenna elements are strategically distributed with an inter element spacing of approximately 0.24 λ (λ being wavelength related to operating frequency). Table 1 displays the dimensions for the irregular octagon two-port MIMO antenna proposed here. Notably, the proposed textile antenna’s dimensions are compact, measuring 40 mm by 50 mm, demonstrating enhanced efficiency. Extending a two-port wearable antenna design to include more ports requires careful consideration of various factors, including coupling, geometry optimization, matching networks implementation, feeding network setup, simulation testing, and comprehensive simulation/testing.

An equivalent circuit for a patch antenna provides an accurate representation of its electrical properties in an easily understood form, featuring components like capacitors, inductors, and resistors to model its behavior and understand and analyze its performance, such as impedance matching, radiation pattern, and bandwidth characteristics, without complex electromagnetic simulations.

**Figure 2:**
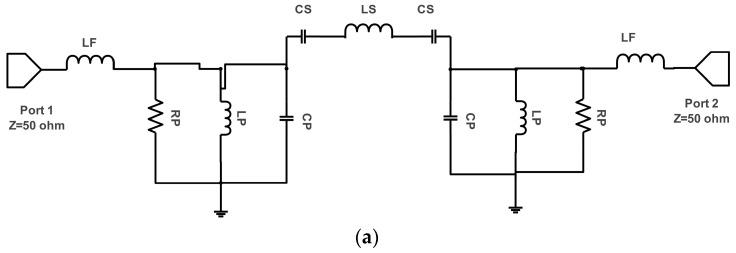

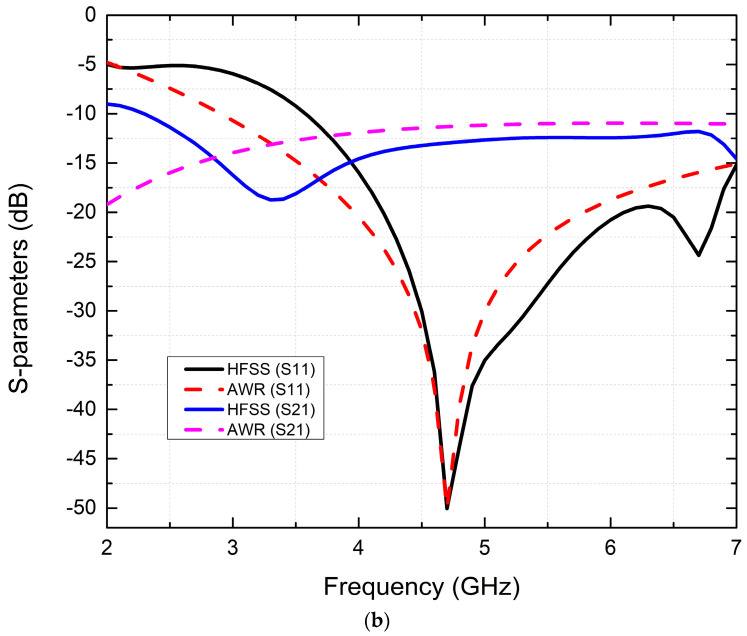
Proposed antenna. (**a**) Equivalent circuit. (**b**) S-parameters.

The AWR Design Environment allows users to quickly determine the equivalent circuit parameters of an antenna by selecting its components. The patch antenna with a series inductor with an initial value of 0.01 pF is connected to a parallel RLC patch circuit featuring extracted values Rp = 54 Ω, Lp = 8 nH, and Cp = 0.33 pF. Additionally, the CLC circuit model for parasitic elements with inductance extracted as Ls = 4.42 nH and Cs = 16.85 pF between antenna elements and the parasitic element [25] is shown in Figure 2a. Differences between both S-parameters in the simulation are accepted and reasonable between the simulation and model circuit simulation models in terms of both model S-parameter values, as shown in Figure 2b.

## 3. Bending Analysis

Bending analysis of a flexible jeans-based antenna involves a meticulous examination of the antenna’s behavior when subjected to various curvatures or bends. This evaluation is pivotal in assessing the antenna’s performance, durability, and signal integrity in real-world scenarios. From an electromagnetic perspective, the bending analysis delves into how curvature affects the antenna’s radiation pattern, resonant frequency, bandwidth, and impedance matching. Moreover, the resonance frequency of the antenna can experience deviations as the curvature induces variations in the electrical length of the radiating element.

**Figure 3:**
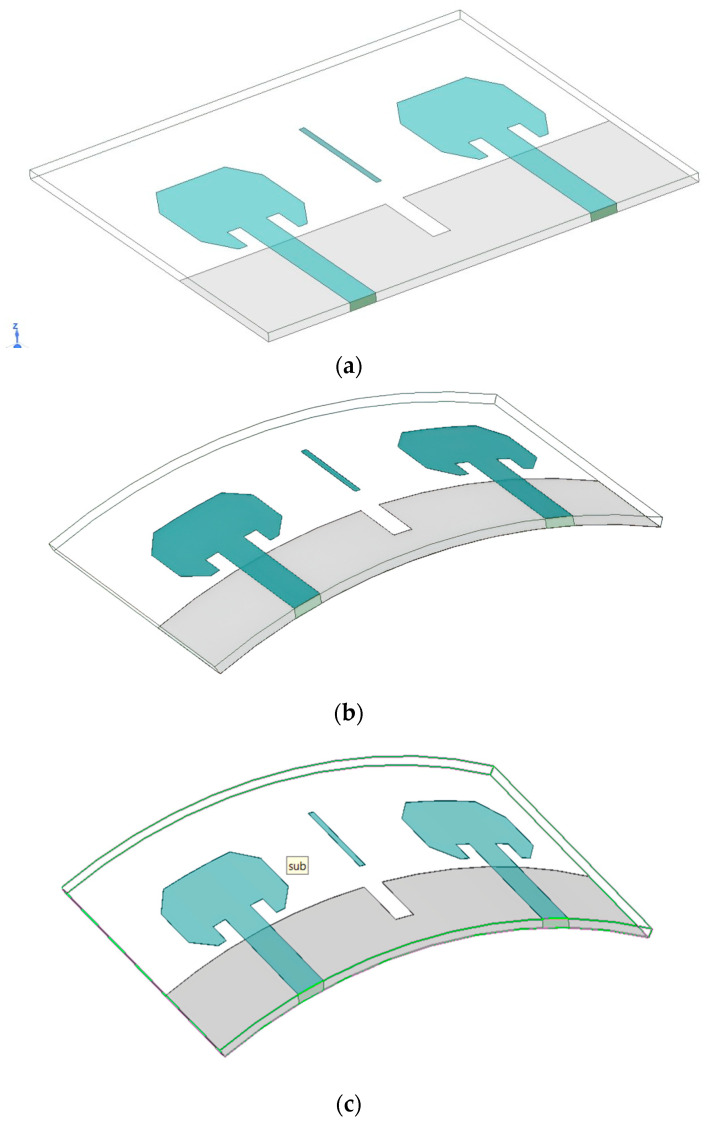
Bending analysis of the antenna.(**a**) Theta = 0 (i.e., flat). (**b**) Theta = 40. (**c**) Theta = 45.

**Figure 4:**
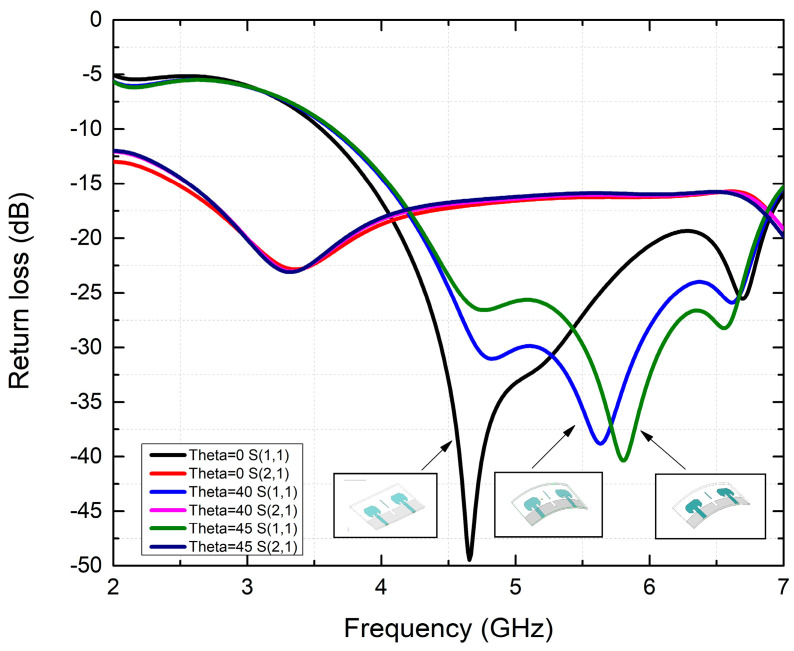
Comparison of the S-parameters of bending analysis.

Figure 3 depicts the flexible configuration of the antennas under consideration in case (i) R = 60, theta = 40, and case (ii) R = 50, theta = 45. While the impedance matching remains consistent during operation, the resonance frequency experiences a slight rightward shift owing to the curvature of the structure. Notably, even with further bending, the antennas consistently retain their higher frequency band across all scenarios. In conclusion, the impedance, resonance frequency, and bandwidth of the novel bent antenna exhibit strong performance, underscoring its suitability for real-world communication scenarios. Figure 4 depicts three scenarios concerning S-parameters.

## 4. Results

**Figure 5:**
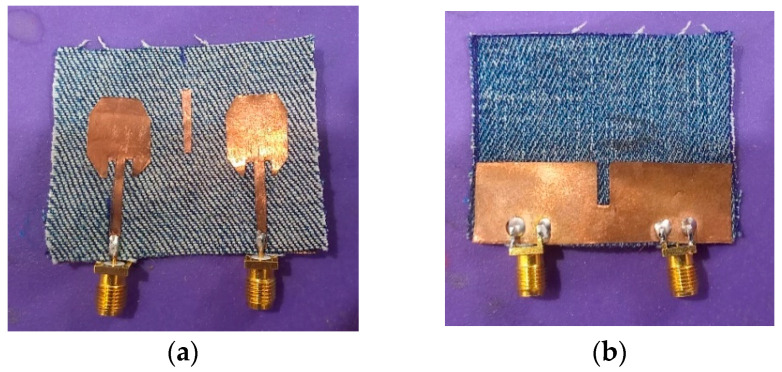
Proposed textile irregular octagon antenna. (**a**) Top view. (**b**) Bottom View.

**Figure 6:**
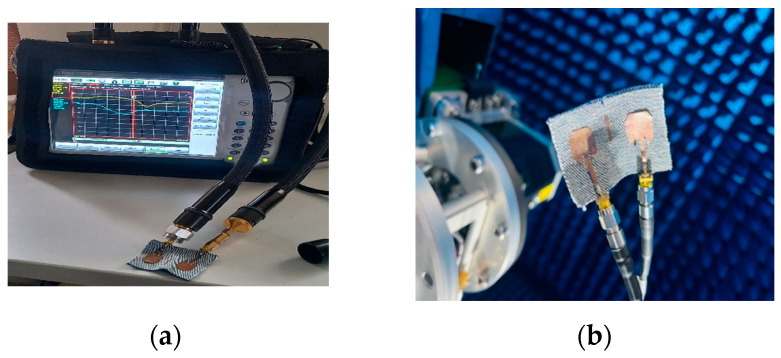
Proposed textile irregular octagon antenna measurement setup. (**a**) VNA 5, (**b**) anechoic chamber.

Figure 5 displays a prototype of a proposed textile two-port irregular octagon MIMO antenna design. It was constructed on a substrate composed of jeans textile and copper foil with thicknesses of 1 mm and 0.07 mm, respectively. Modeling for performance assessment using the finite element method (FEM) in HFSS software v21 was then conducted while measurements using an Anritsu Model: S820E MS2038C Vector Network Analyzer were performed within an anechoic chamber as demonstrated in Figure 6. Section 3 provides an analysis of the bending characteristics that the proposed antennas possess and conducts an experiment to verify them with slightly bending wearable antenna; this test validates their bending properties and further establishes their effectiveness within their operating ranges.

### 4.1. Reflection Coefficient Results

**Figure 7:**
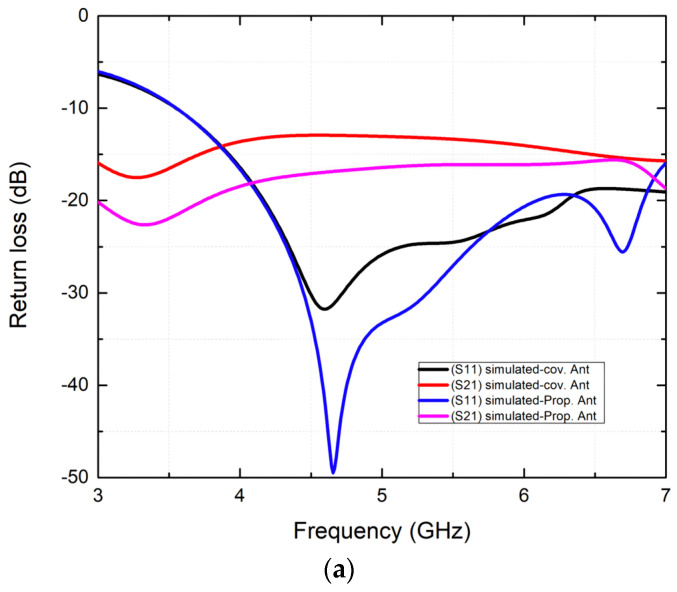

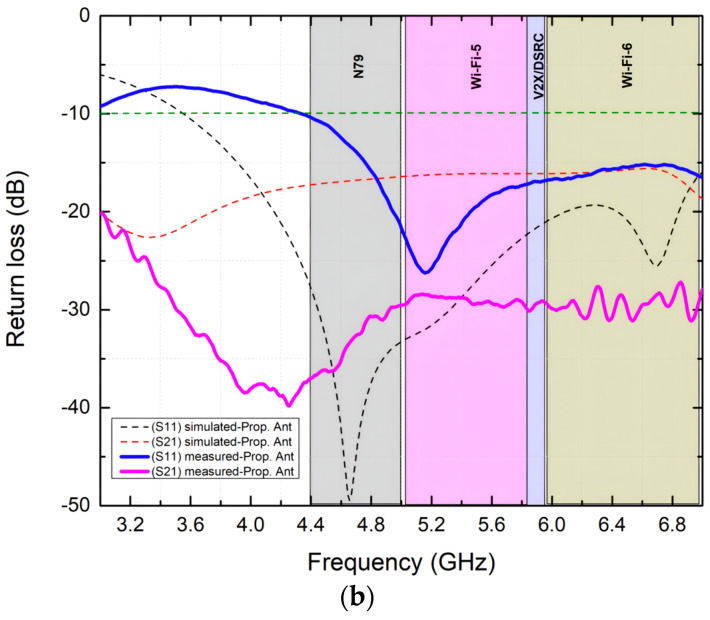
S-parameter plot of simulated and measured results for the (**a**) conventional and (**b**) proposed antenna.

Figure 7a shows the reflection and transmission coefficients of the conventional two-port antenna (i.e., an irregular octagonal antenna) and the proposed two-port irregular octagon-shaped wearable antenna. In Figure 6b, the measured impedance bandwidth is illustrated, showing values of 45.6% (4.35–7 GHz) for the 5G NR mid-band sub-6 GHz. During the HFSS simulation, simulated results achieved an impedance bandwidth of 64.15% (3.6–7 GHz). However, the HFSS simulation indicated some minor variations. Moreover, the obtained results exhibit minor deviations attributed to fabrication errors and conductor loss in cables encountered during the measurement analysis, as depicted in Figure 7b. The simulated and measured isolation parameter provides good isolation between the proposed two-port flexible MIMO antennas. The measured lowest isolation is found at −28 dB and it is maintained over the entire sub-6 GHz band. The wearable jeans textile irregular octagon-shaped two-port MIMO antenna covers sub-6 GHz frequencies includes the N79 band (4.40–5 GHz), the Wi-Fi-5 band (5 GHz to 5.85 GHz), the V2X/DSRC bands (585–590 GHz) and the Wi-Fi-6 band (5.925–7.125 GHz) for 5G communications. Due to the symmetry of this two-port antenna design, only single antenna parameters are shown, and this information applies directly to the other antenna as well. A similar antenna design was tested successfully within [2], further validating its use. Comparing on-body effects with off-body results revealed no deviation from the intended frequency bands, confirming the reliability of this method. HFSS v21 simulation software enabled the thorough examination of wearable antennae’s characteristics as well as a precision equivalent to testing conducted on human beings for accurate and scientifically valid results.

### 4.2. Surface Current Distribution

**Figure 8:**
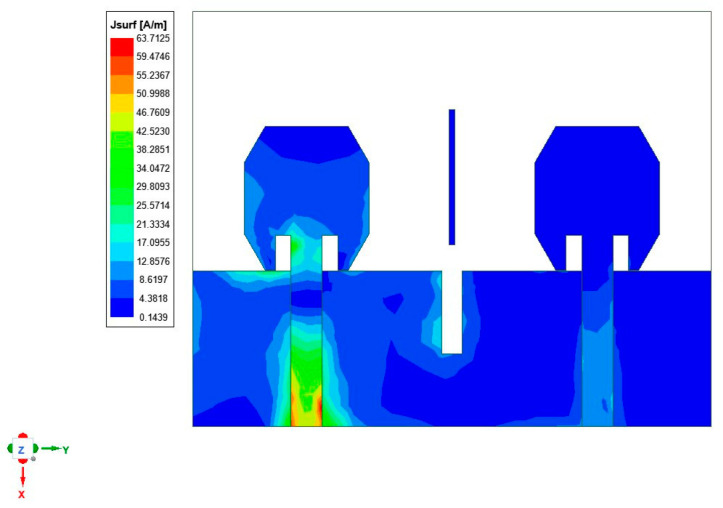
Current distribution at a frequency of 4.6 GHz.

At an operating frequency of 4.6 GHz, an irregular octagon-shaped wearable MIMO antenna was subjected to testing of the current distortion. Figure 8 depicts the current distribution and illustrates the coupling effect of the current between the proposed antennas and the elements. When a slot is inserted in the ground, it alters the current distribution between the ground planes, and the maximum current density is concentrated at the slot to cancel the radiated and induced fields. The parasitic strip helps to achieve good impedance matching and isolation between antenna elements. The combination of a slot within the ground plane, as well as an extra strip of the parasitic element in between the radiating components, was essential in achieving isolation and impedance matching simultaneously. Hence, the isolation level is improved by −12 dB.

### 4.3. Radiation Properties

**Figure 9:**
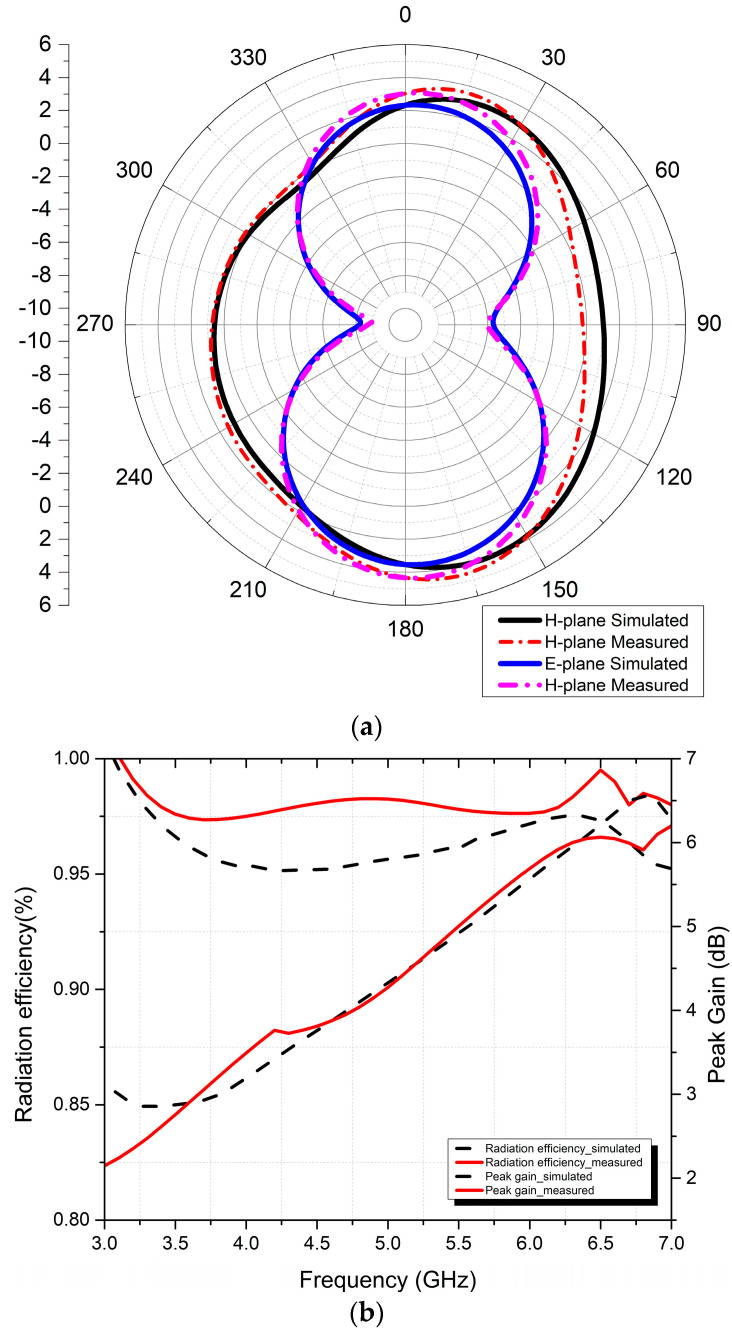
Two-port irregular octagon antenna (**a**) radiation pattern and (**b**) gain and efficiency.

In Figure 9, the proposed irregular octagonal two-port textile MIMO antenna’s radiation performance in both the E and H planes is examined in depth. Figure 9a shows 2D radiation patterns calculated at 5.2 GHz according to both the measured and simulated results; these two-dimensional plots reveal that the antenna is radiating omnidirectionally. Notably, both the measured and simulated radiation patterns demonstrate high degrees of agreement in the E and H planes, respectively. 

Figure 9b illustrates both the simulated as well as the measured gains and radiation efficiency of the irregular octagonal two-port textile MIMO antenna across frequencies from 3 GHz to 7 GHz. Our wearable textile irregular octagon-shaped two-port MIMO patch antenna exhibits gains of up to 4.1 dB at frequencies as low as 4.6 GHz, with maximum gains reaching 6.5 dB, and the efficiency is 96% when set to 6.75 GHz.

### 4.4. SAR Calculation

To better understand how radiation impacts our bodies, HFSS has integrated an anatomically accurate body model into its antenna array for the easy simulation analysis of its effects. This configuration enables simulations of radiation’s potential effects. The SAR value was determined using Equation (2).

(2) $$SAR=\sigma \frac{{\left|E\right|}^{2}}{\rho }$$

**Figure 10:**
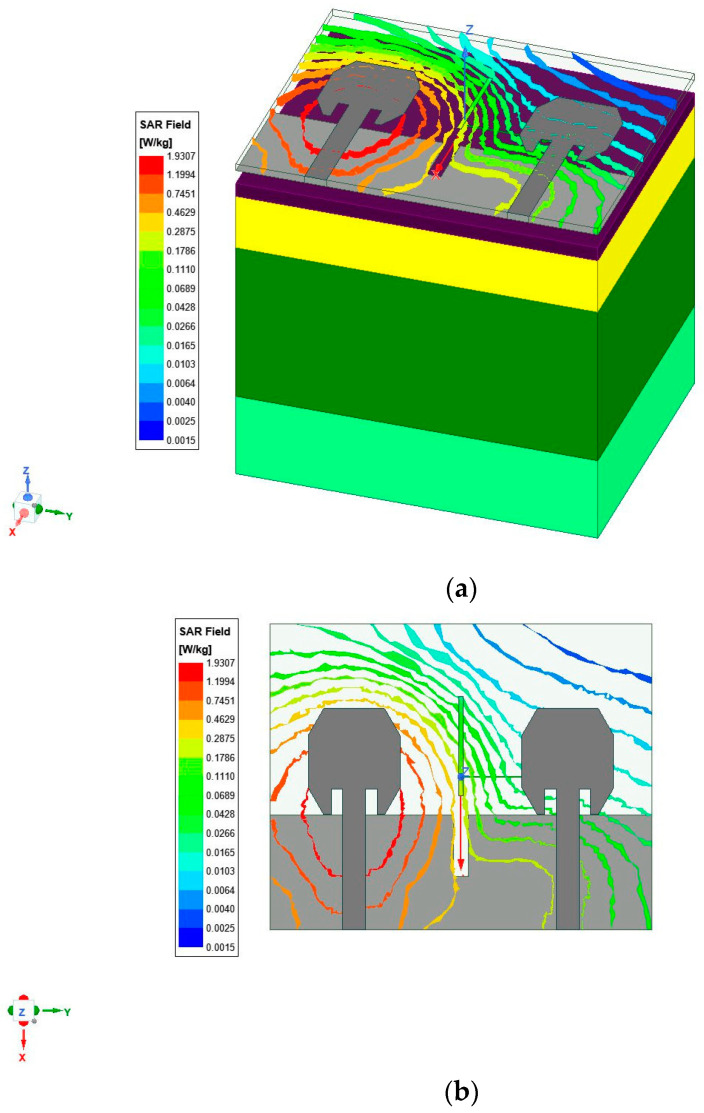
Irregular octagonal patch antenna SAR analysis on (**a**) a human phantom and (**b**) an antenna.

Here, the σ, E and ρ refer to conductivity (s/m), field intensity (V/m), and the density of biological tissue as a measure of mass in Kg/m^3^. Below, Table 2 lists the properties of tissues of the human body:

Because antennas emit backward radiation, it is imperative to assess their specific absorption rate (SAR). To achieve this, a flat phantom representing body wear is used, in line with the SAR research methodology; antennas are then mounted approximately 2 mm above this human phantom. Figure 10a depicts cross-sectional views of human three-layer models consisting of skin, fat, and muscle layers, while Figure 10b presents SAR analysis conducted on proposed textile irregular octagonal patch MIMO antenna when placed onto human bodies.

## 5. MIMO Antenna Diversity Parameters

### 5.1. Envelope Correction Coefficient

Evaluating ECC in our MIMO antenna system is integral for measuring its diversity performance, with ideal ECC values being below 0.5, and measuring how effectively this prototype provides robust diversity based on this threshold. ECC measures isolation and correlation across communication channels, as expressed by Equation (3).

(3) $$\rho =\frac{{\left|S_{11}^{*}S_{12}+S_{21}^{*}S_{22}\right|}^{2}}{\left(1-{\left|S_{11}\right|}^{2}-{\left|S_{12}\right|}^{2}\right)\left(1-{\left|S_{21}\right|}^{2}-{\left|S_{22}\right|}^{2}\right)}$$

**Figure 11:**
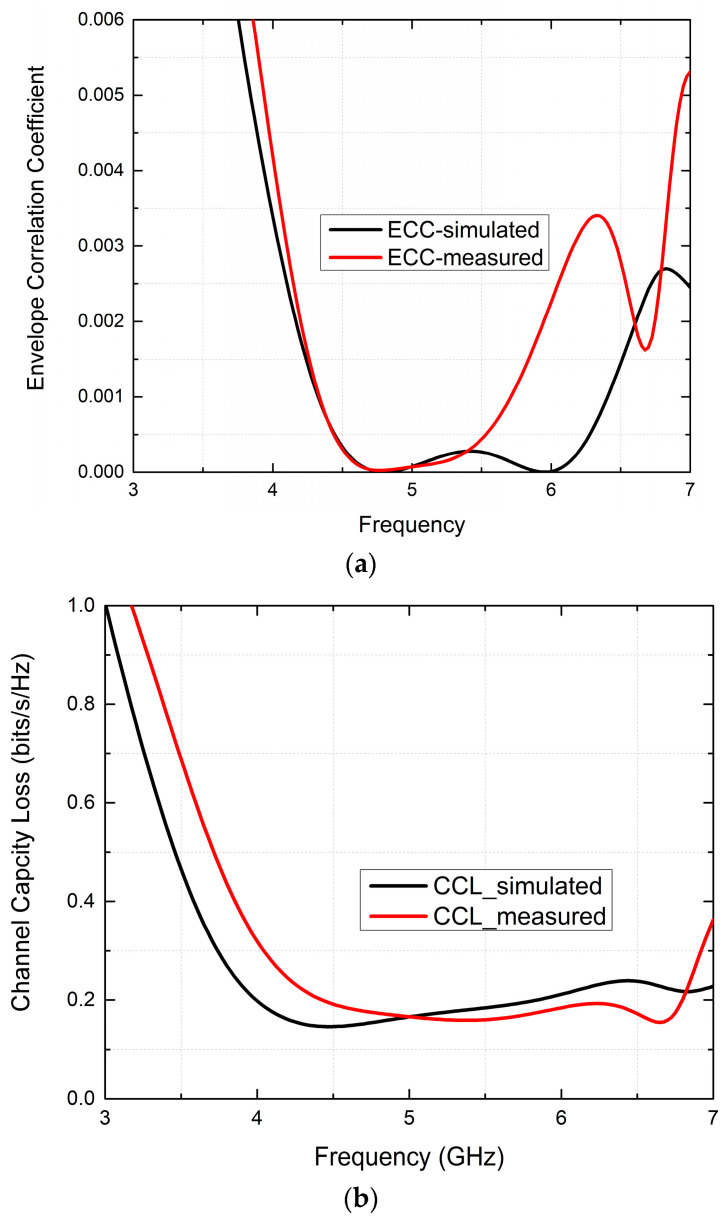
The proposed antenna diversity parameters. (**a**) ECC. (**b**) CCL.

The determination of the error correction code (ECC) for the envisioned MIMO system involves the utilization of the measured S-parameters. The ECC curve in Figure 11a distinctly reveals that the ECC value consistently maintains a level below 0.025 throughout the entirety of N79, Wi-Fi-5/6, and V2X/DSRC in the sub-6 GHz band. This finding confirms that the prototype excels in multiplexing performance, ultimately boosting data throughput. This achievement holds significant importance in contemporary communication systems.

### 5.2. Channel Capacity Loss

CCL is a crucial metric that plays a vital role in assessing MIMO channel capacity, serving to quantify correlation among closely spaced patch elements within MIMO systems. When working in multipath environments with a high signal-to-noise ratio, its calculation requires using Equation (4a).

(4a) $$C_{loss}=-{log}_{2}^{\left|\Psi^{R}\right|}$$

Assuming that $\Psi^{R}$ represents an *n* × *n* correlation matrix (i.e., *n* = 2), components of MIMO systems can be described by using one of the three equations shown here, and are expressed relative to S-parameters in Equations (4b)–(4e).

(4b) $$\Psi_{11}=1-{\left|S_{11}\right|}^{2}-{\left|S_{12}\right|}^{2}$$

(4c) $$\Psi_{22}=1-{\left|S_{21}\right|}^{2}-{\left|S_{22}\right|}^{2}$$

(4d) $$\Psi_{12}={-(S}_{11}^{*}S_{12}+S_{21}^{*}S_{22})$$

(4e) $$\Psi_{21}={-(S}_{22}^{*}S_{21}+S_{12}^{*}S_{11})$$

In MIMO systems, the primary objective is often to achieve a very low CCL to maximize channel capacity. For the specific MIMO antenna under consideration, the CCL threshold is established at 0.4 bits/s/Hz. As depicted in Figure 11b, the CCL consistently maintains a significantly low value (CCL < 0.13 bits/s/Hz) throughout the overall operating band. Consequently, the proposed irregular octagon-shaped MIMO antenna proves to be well-suited for applications in wearable systems.

## 6. Comparison of Existing Systems and Proposed Antenna

Table 3 provides a comparison between various parameters of different antennae, including size, frequency range, gain, and isolation, including the proposed system and existing systems.

Regarding the existing MIMO antennas for portable wireless applications with specific outcomes, each is different from the others in terms of its size or operating frequency range. If we focus on one particular antenna parameter, then we might not even obtain ideal values for other antenna parameters. There is an existing MIMO antenna that has dimensions of 40 × 70 mm and the gain achieved by this antenna is 4.4 dB in the sub-6 GHz band from 4.4 GHz to 8 GHz. Here, we propose a MIMO antenna with dimensions of 40 × 50 mm^2^, and we achieved 6.74 dB of gain, which is greater than the average gain and provides better return loss, at around −45 dB, and better directivity. With regard to antenna size, this antenna is smaller, providing improved results within its target frequency range of approximately 4.35 GHz to 7 GHz, making it well suited to 5G applications.

## 7. Conclusions

This paper introduces an innovative MIMO antenna system designed to deliver superior isolation and stable pattern characteristics suitable for wearable applications. This antenna features a single conducting layer in the shape of an irregular octagon and can be seamlessly integrated into wearable jeans. The design process is employed in an FEM-based HFSS full-wave simulator. The compact MIMO antenna, measuring 50 mm × 40 mm, was physically fabricated on 1 mm^2^ of jeans cloth material. This antenna system operates across a wide bandwidth in sub-6 GHz, offering a fractional bandwidth of 45.6%, and has been demonstrated to withstand bending and body movement without compromising its performance. The antenna system achieves gain, radiation efficiency, and SAR values of 6.7 dB, 96%, and 1.93, respectively. The gains achieved by the proposed antenna system ensure robust and dependable communication links for wearable devices operating within the sub-6 GHz band, thus providing seamless connectivity for diverse applications for the N79, Wi-FiFI-5, Wi-Fi-6, and V2X/DSRC bands.

## Figures and Tables

**Table 1:** Dimensions of the irregular octagon-shaped antenna.

Parameters	Value (mm)	Parameters	Value (mm)	Parameters	Value (mm)
W	50	L	40	Lg	25
P1	8	P2	12	P3	1.5
P4	3.35	P5	3	P6	7
Sw	2	SL	8	W0	25
Pw	0.6	PL	13	P7	18.43
P8	10.5	D1	4	D2	4
D3	4	D4	4		

**Table 2:** Properties of tissues of the human body.

Tissue Type	Skin Layer	Fat Layer	Muscle Layer	Bone
Permittivity $\left(\varepsilon_{r}\right)$	42.1	5.3	52.8	18.5
Conductivity (S/m)	1.6	0.1	1.7	0.83
Loss tangent	0.28	0.15	0.25	0.26
Density (Kg/m^3^)	1108	910	1092	1010
Thickness (mm)	2	8	23	12

**Table 3:** Comparison of existing systems and the proposed antenna.

Reference	Dimensions (mm^2^)	Dielectric Permittivity (ε_r_)	Operating Frequency (GHz)	Peak Gain (dB)	Isolation (dB)
[2]	40 × 70	1.6	2.4–8.0	4.4	Min 22 Max. 53
[8]	30 × 30	10.2	2.37–2.52	-	>20
[9]	38.1 × 38.1	4.4	2.3–2.8	2.79	12
[10]	92.3 × 101.6	1.3	2.367–2.53 5.147–5.863	5.8	20 35
[11]	40 × 40	1.7	3.3–3.6 4.5–5	-	>17
In this paper	40 × 50	1.6	3.6–7 (simulated) 4.35–7 (measured)	6.5	>12 >28.5

## Data Availability

The data will be provided on the request basis.
